# Beta-Lactam Antibiotics as A Possible Novel Therapy for Managing Epilepsy and Autism, A Case Report and Review of Literature 

**Published:** 2015

**Authors:** Ahmad GHANIZADEH, Michael BERK

**Affiliations:** 1 Research Center for Psychiatry and Behavioral Sciences, Shiraz University of Medical Sciences, Shiraz, Iran; 2 Department of Psychiatry, Shiraz University of Medical Sciences, School of Medicine, Shiraz, Iran; 3 Department of Neuroscience, School of Advanced Medical Sciences and Technologies, Shiraz University of Medical Sciences, Shiraz, Iran.; 4 Professor of Psychiatry, School of Medicine, Deakin University, Melbourne, Australia; 5 Professorial Research Fellow, The Florey Institute of Neuroscience and Mental Health, Orygen Research Centre and the Department of Psychiatry, University of Melbourne, Melbourne, Australia

**Keywords:** Cefixime, Antibiotic, Glutamate, Transporter, Therapy, Inflammation

## Abstract

Autism is a disorder of unknown etiology. There are few FDA approved medications for treating autism. Co-occurring autism and epilepsy is common, and glutamate antagonists improve some symptoms of autism. Ceftriaxone, a beta-lactam antibiotic, increases the expression of the glutamate transporter 1 which decreases extracellular glutamate levels. It is hypothesized that modulating astrocyte glutamate transporter expression by ceftriaxone or cefixime might improve some symptoms of autism. This case report of a child with autism and epilepsy suggests a decrease in seizures after taking cefixime

## Introduction

Autism is a complex disorder marked by impairment of verbal communications, social relationship problems, repetitive behaviors, and restrictive interests. Its etiology is unclearly understood and there is debate about its pathogenesis. There are no effective therapies for autism. Novel therapeutic interventions for treating autism are therefore a priority. Autism is additionally highly comorbid with epilepsy (OR=22.2; 95% CI=16.8-29.3) (1), but treatment refractory epilepsy in autism occurs in up to 33.9% of individuals (2). Complicating this, outcomes of surgical and vagus nerve stimulator (VNS) implantation in patients with both autism and epilepsy are less effective than in other treatment refractory epilepsy patients (2). 

Glutamate is a crucial neurotransmitter in the brain. It is released from cells into the extracellular fluid and then removed by glutamate transporters. This transportation regulates excitatory synaptic transmissions. While the level of glutamine is decreased, glutamate level is increased in autism and tryptophan in autism (3). 

The excitatory amino acid transporter of EAAT2 (or GLT1) is one of the main glutamate transporters in brain. About 90% of glutamate transporters in brain are of the EAAT2 type (4). Excitatory amino acid transporters keep the extracellular glutamate level lower than the neurotoxic level, and GLT-1 is the major determinant of glutamate level in extracellular fluid. The decreased expression and function of astrocyte glutamate transporters enhances the levels of extracellular glutamate in epilepsy and Tuberous Sclerosis Complex (5). Tuberous Sclerosis Complex is usually co-morbid with epilepsy and autism. Astrocyte glutamate transporters are deficient in animal models of epilepsy in Tuberous Sclerosis Complex (6). This abnormality is also reported in human and animal models of epilepsy. In addition, targeting astrocytic mechanisms has been introduced as a novel treatment approach for epilepsy and TSC (6).

The beta-lactam antibiotics cefixime and ceftriaxone are third-generation cephalosporins. Ceftriaxone increases GLT1/EAAT2 expression (7). Cefixime and ceftriaxone are beta lactam antibiotics, and the latter improves the expression of glutamate transporter (GLT-1). Glutamate neurotoxicity may contribute as a pathological mechanism for autism (8). There is an abnormal GABA to glutamate ratio (8) suggesting an imbalance of inhibitory and excitatory systems in the neurobiology of autism (9). Glutamate synthesis inhibition attenuates neurotoxic activity in Rett’s syndrome (10). It is suggested that attenuation of the hyperglutaminergic state in autism may improve symptoms (11). Equally, up regulation of the GABAergic system is proposed to have utility (11, 12). These transporters play an important role in vitro and in vivo for preventing of glutamate neurotoxicity. Ceftriaxone decreases extracellular glutamate levels by the enhancement of astrocyte glutamate transporters expression (6). In addition, ceftriaxone has low toxicity. A recently published study showed that ceftriaxone is neuroprotective during the acute phase of ischemia (13). 

Antagonism of glutamate receptors has been suggested as a potential mechanism for the treatment of autism. However, regulating glutamate transporter expression and activity has an advantage of minimizing the pathological impact of glutamate overload while maintaining a physiological role of glutamate. It is unclear whether the extracellular glutamate transporter level is decreased in the disorder. 

## Case presentation

The index patient is a 9 year old boy with autism spectrum disorder diagnosed according to Diagnostic and Statistical Manual of Mental Disorders (DSM-IV). He suffered from generalized tonic-clonic epilepsy from age 4. He had taken multiple different medications such as phenobarbital, sodium valporate, and carbamazepine with sufficient dosages and durations without favorable control of his epilepsy. According to his parents’ reports, the patient took cefixime 200mg/day to control diarrhea about 2 years ago. The seizure episodes were dramatically decreased 3 days after starting the medication while the there was no change in his anti-epileptic medication regime. The seizure episodes were controlled for about 5 months, after which the number of seizure episodes again increased. His highly educated parents administered cefixime 200mg/day to control seizure again. They reported that seizure attacks were controlled markedly after taking cefixime for three days. The patient was not febrile while the medication trials were administered. Both parents reported that they repeated this trial for several times to control the seizure episodes in the recent years. The epilepsy was controlled in all of the trials after taking cefixime for 3 to 5 days. Then, they discontinued cefixime after 7 days. They reported that there was a marked decreased in the number of seizure attacks as well as aggressive behaviors. Physical examination did not show any remarkable finding such as fever, headache, or photophobia. Brain MRI did not illustrate any abnormal finding. No remarkable finding was found on laboratory examinations. This is a retrospective case report, and caution needs to be used in interpreting case reports. His parents provided their consent for publication of this report. 

## Discussion

Neuroinflammation is proposed to contribute to the neurobiology of both autism and epilepsy (14-17). Recently, anti-inflammatory and immunosuppressive drugs have showed promising therapeutic effects in autism (18) and epilepsy. For example, some antibiotics including doxycycline, minocycline or tetracycline protect against seizures in experimental seizure models in rats and mice (19). However, Clavulanic acid, which inhibits bacterial b –lactamases did not affect convulsions in acute seizure tests in mice (20). 

In several trials, cefixime was administered without any co-administered medication. Therefore, drug interaction was not a likely explanation for this association. A few days of seizure control cannot be attributed to a short course of cefixime but the patient experienced several consequent episodes of seizure before administering cefixime. After administering cefixime, these seizure episodes stopped. Whenever cefixime was not administered, seizure episodes happened again. 

Seizures induce brain inflammation and increases interleukin (IL)-1β, potentially disrupting the blood brain barrier (21). Some antibiotics such as minocycline may decrease the epileptic seizure through anti-inflammatory effects (22), and this effect is a further possible explanation for the effect of cefixime on epilepsy. 

In view of the limited treatment approaches for autism, the proposed role of glutamate in pathophysiology of autism (9), the promising efficacy of glutamate receptors antagonism for the treatment of autism (23), the role of excitatory amino acid transporters in keeping extracellular glutamate level below neurotoxic levels and the increased expression of the glutamate transporter (GLT-1) by ceftriaxone (7), it seems to be reasonable to hypothesize that modulating astrocyte glutamate transporter expression by ceftriaxone or cefixime might be at least partially effective for treating autism and epilepsy. Overexpression of vesicular glutamate transporter levels in Drosophila causes excess glutamate release which leads to excitotoxicity. As the level of glutamate is increased in autism, it is proposed as a viable target for treating autism. Therefore, targeting glutamate by beta lactam antibiotics might be effective for treating both autism and seizures. Well controlled clinical trials are needed to examine the effects of beta lactam antibiotics on animal models of autism plus epilepsy. Cefepime, a fourth-generation cephalosporin antibiotic, is associated with seizure risk in some reports (24). Future studies should use more objective measures such as EEG monitoring and blood drug concentration assessment. Consequently, this case report should be considered as preliminary and hypothesis generating. 
